# Emergency Response to COVID-19 in Canada: Platform Development and Implementation for eHealth in Crisis Management

**DOI:** 10.2196/18995

**Published:** 2020-05-15

**Authors:** Michael Krausz, Jean Nicolas Westenberg, Daniel Vigo, Richard Trafford Spence, Damon Ramsey

**Affiliations:** 1 Department of Psychiatry University of British Columbia Vancouver, BC Canada; 2 Department of Surgery University of Toronto Toronto, ON Canada; 3 InputHealth Systems Inc Vancouver, BC Canada; 4 Department of Family Practice University of British Columbia Vancouver, BC Canada

**Keywords:** eHealth, digital health, web-based intervention, crisis management, COVID-19, public health, health care system

## Abstract

**Background:**

Public health emergencies like epidemics put enormous pressure on health care systems while revealing deep structural and functional problems in the organization of care. The current coronavirus disease (COVID-19) pandemic illustrates this at a global level. The sudden increased demand on delivery systems puts unique pressures on pre-established care pathways. These extraordinary times require efficient tools for smart governance and resource allocation.

**Objective:**

The aim of this study is to develop an innovative web-based solution addressing the seemingly insurmountable challenges of triaging, monitoring, and delivering nonhospital services unleashed by the COVID-19 pandemic.

**Methods:**

An adaptable crisis management digital platform was envisioned and designed with the goal of improving the system’s response on the basis of the literature; an existing shared health record platform; and discussions between health care providers, decision makers, academia, and the private sector in response to the COVID 19 epidemic.

**Results:**

The Crisis Management Platform was developed and offered to health authorities in Ontario on a nonprofit basis. It has the capability to dramatically streamline patient intake, triage, monitoring, referral, and delivery of nonhospital services. It decentralizes the provision of services (by moving them online) and centralizes data gathering and analysis, maximizing the use of existing human resources, facilitating evidence-based decision making, and minimizing the risk to both users and providers. It has unlimited scale-up possibilities (only constrained by human health risk resource availability) with minimal marginal cost. Similar web-based solutions have the potential to fill an urgent gap in resource allocation, becoming a unique asset for health systems governance and management during critical times. They highlight the potential effectiveness of web-based solutions if built on an outcome-driven architecture.

**Conclusions:**

Data and web-based approaches in response to a public health crisis are key to evidence-driven oversight and management of public health emergencies.

## Introduction

Health care systems development is determined by existing structures and traditions, but they also change rapidly in response to crises, public health threats, or urgent societal needs. The 2009 influenza pandemic and the 2014 Ebola virus disease outbreak had a global impact and revealed gaps in the structure of health care systems in countries around the world [1,2]. Epidemics have always been a time of enormous challenges, causing preventable fatalities that uncover structural and interventional deficits in the system—the coronavirus disease (COVID-19) pandemic is no different. Severe acute respiratory syndrome coronavirus 2 was first reported in Wuhan, Hubei Province, China and has subsequently spread at an alarming rate, becoming a global pandemic and provoking a shutdown for which we were all ill-prepared [3].

Large-scale community containment efforts have been deployed in several countries in an attempt to “flatten the curve,” but health care systems are still falling short. In Italy, Spain, and the United States, health care workers are failing to meet the high demand, and patients in critical condition are saturating the intensive care units [4]. In the United Kingdom, the critical care bed demand is expected to be exceeded, with an eventual peak in intensive care unit demand that is over 30 times greater than the maximum supply, despite mitigation strategies [5]. As more cases appear, frontline health care workers are under pressure and called into action at the detriment of their own safety [6,7].

Challenges in reorganizing care during a system crisis are significant. Response teams need and deserve a continuous flow of accessible data to respond effectively to the dynamic of an epidemic. Moreover, the interaction with patients and potentially their social network is critical for the efficacy of crisis response—empowering and engaging patients into any approach can make all the difference [8]. Online communication can facilitate and organize patient involvement while collecting essential data to facilitate efficient use of finite human and nonhuman health care resources [8]. Ideally, such patient interfacing would be set prior to any immediate need of crisis management such as with the current pandemic.

As identified throughout the literature and in experience, major shortcomings in health care crisis management systems can be summarized as the following:

1. Lacking centralized and intelligent screening and triaging [9]: current centralized services that offer medical advice and health information, such as telephone support lines, are not built to scale nor can they handle surges in demand. Although a plethora of single-use screening tools, whether online or not, have been deployed, these tools tend to operate disparate from the existing health care system, do not integrate to primary care, and do not offer intelligent risk stratification that can adapt to changing needs of a pandemic.
2. Difficult referral management and absence of integrated surveillance [10]: rapidly evolving dynamic circumstances require rapid reallocation of health care resources such as health care workers and medical devices based on prioritization. Patient and health care delivery are impacted by existing referral management systems, which lack any meaningful “forward triage.” There is no centralized and scalable system able to provide ongoing management of patients and health care delivery, as well as surveillance of the public for symptoms and risk factors.
3. Lack of capacity to seamlessly deliver care [10]: existing tools tend to simply screen or deliver information, rather than provide a seamless pathway to actually deliver care, such as through asynchronous messaging, instant messaging, and video and audio communication tools. The predominant communication methodologies leave patients in limbo and contribute to confusion and a potential worsening of the crisis through a lack of patient flow control.
4. No built-in analytic engine: there is a lack of live visualizations and reporting of data that is collected securely and in real time, leaving little opportunity to act or improve.

All of these points are exaggerated in the context of an infectious pandemic in proportion to the enormous pressure on individuals and systems. Structural and governance problems as well as any existing dysfunctional processes in clinical and logistics pathways become visible at once. The lack of preparedness is notable worldwide, which, among other things, generates lack of confidence and fear in the public. When systems are ineffective and ill-prepared, solutions to address crises can become multiple, fragmented, and ineffective.

The sudden increased demand on delivery systems puts unique pressures on pre-established care pathways. These extraordinary times require efficient tools for smart governance and resource allocation. Web-based solutions in health care represent a paradigm shift in communication and organization of health care [9,11]. Critical to their scalability and interoperability is their architecture and the clinical principles they translate into practice. They are a tool that reflects the underlying treatment and care philosophy [8,12,13].

The objectives of this paper are to describe the obvious needs that must be addressed in the crisis response to an upcoming epidemic and the conceptional framework for a web-based solution addressing these problems. As part of a public health response, we aim to make the design and development process transparent and accessible for further evaluation. The strength of web-based solutions in this context will be reflected in the presentation of a specific solution developed in response to COVID-19. This specific solution will incorporate the functionalities needed to better respond to the needs of the population.

## Methods

In response to the COVID-19 pandemic, we hypothesized that web-based solutions represent a paradigm shift in communication and organization of health care, which could address the shortcomings of traditional crisis management systems to manage an infectious pandemic**.**

We reviewed the current literature on existing web-based solutions and consulted with medical specialists, decision makers, and policy makers to address the major shortcomings in the current crisis management systems. We then conceptualized how web-based solutions could address these shortcomings.

Using the conceptual framework, we endeavored to develop a web-based, lightweight, and cloud-based crisis management system designed for rapid deployment. As COVID-19 gives new urgency to a long-neglected demand for change in health care paradigms, this crisis management system was aimed to have health care meet people where they are, instead of bringing people to where health care is. The design of such a solution was driven by the need for an urgent, scalable, and efficient set of deliverables, regardless of setting or method. An ideal response would be a rapidly implementable and scalable method for mass screening and continuous monitoring for potentially the majority of the world’s population. Only web-based tools can be deployed so rapidly and scaled up to large areas and populations with relatively minimal marginal cost.

## Results

### Conceptual Framework

The conceptual framework for a crisis management system that addresses the major shortcomings identified includes the following:

1. Centralizing the screening and triage process with a single, shared platform that integrates primary care: screening can happen automatically at scale without absorbing resources and can take place in a manner that is both standardized as well as agile to accommodate the changing screening criteria. Based on data collected from patients, there is an opportunity to triage patients to ensure they are directed to the most appropriate type of care (eg, self-isolation, primary care, emergency department). A cloud-based solution could unite health care providers across the region, providing a single platform to deliver care for patients during a pandemic while also allowing individuals to connect to their own primary care provider where possible. This counteracts substantial silos that exist presently with disparate systems that produce barriers to collaboration.
2. Automated ability to track and follow up with patients: A built-in remote monitoring capability via symptom-tracking questionnaires with automatic alerts would enable providers to manage the majority of patients in their own homes, addressing patient and provider health as well as source control.
3. Integrated and intelligent virtual care: a web-based solution has the ability to provide virtual care and self-management strategies directly through the platform, including secure video, audio, and instant messaging. This can increase access by allowing more health care providers to deliver care to patients, reducing personal protective equipment use, and allowing providers to share tools and resources meant to improve the patient’s understanding and improve self-management capabilities.
4. Centralization of data analysis: The combined aggregation of data from screening and triaging as well as clinical data from health care provider interactions and remote symptom tracking could produce a comprehensive data set unlike any other solution. Furthermore, a data analytics engine that allows for real time dashboards of information across the region in a single interface would enable real time responses to a changing pandemic.

Given this conceptual framework, digital health developers designed the Crisis Management Platform (CMP) over a period of 2 weeks. The CMP then had to go through a noncompetitive procurement process run by the provincial government of Ontario, which involved a review of the existing technical, security, and privacy policies and features, before being approved and offered to health authorities in Ontario on a nonprofit basis.

As of the March 23, 2020, the platform was deployed within the province of Ontario, Canada in the London-Middlesex Region and has expanded to other regions including Oxford, Windsor-Essex, Huron, and Perth.

At the time of writing this manuscript, 13,479 patients have been triaged, 401 providers have been onboarded (including 380 medical doctors), and 206 virtual appointments have been conducted [14,15]. As implementation providers are not currently part of our writing group, a follow-up manuscript will describe the experience and review the data in more detail once ethics is established. In the following section, we provide a description of the development and the solution, presented as 6 modules.

### Crisis Management Platform

#### Module 1: Forward Triage Intake

The “forward triage”–oriented CMP initiates patient intake through adaptable branching logic-based questionnaires, which are able to direct or deflect as necessary**.** Initial data collection stratifies patient risk into high, medium, or low risk categories and presents health care access options accordingly based on programable logic: urgent care, emergency department, primary care redirect (Multimedia Appendix 1, page 1). The triage process also supports resource allocation and determines the place in a waiting list or even the immediate necessary crisis response.

Fundamental to the success of the forward triage component of the platform is the ability to rapidly iterate and modify pathways based on new information and data. For example, in the COVID-19 pandemic, it became quickly apparent that travel history was less relevant in screening patient risk when community transmission became predominant. Any platform that is deployed for forward triage must allow for the administrators to control direction and deflection algorithms with ease.

#### Module 2: Autonomous Patient Booking and Registration

Governments across the world, such as in Canada, were quick to release online self-assessment tools for COVID-19. Indeed, such rapid deployment of technology for government was unprecedented. Beyond the press releases, however, is the acknowledgement of fragmented systems where such triage tools have limited end points such as call 911, go to the hospital, talk to your family doctor, or stay at home (with no monitoring available). Furthermore, it became quickly apparent that these tools were not easy to modify, as they continued to propagate questions that were irrelevant based on changing guidelines.

The CMP departed from single use, generic online screening tools by providing an opportunity for patients who were risk categorized appropriately to access same-day appointments (Multimedia Appendix 1, page 2). Rather than ask patients to wait in line, it made abundant sense to allocate appointment slots based on risk category.

#### Module 3: Patient Flow Tracking

A unique innovation in the digital platform is the application of Kanban methodology to patient flow management [16]. Although Kanban was originally designed for manufacturing control in repetitive systems and later adapted more generally to project management, the CMP offers a novel use of the methodology in the management of patient flow. Rather than moving tickets or equipment, the health care provider is able to move patients through customizable clinical pathways, all readily visual through a live-updated online tracking board. Administrators are able to customize the pathways that are possible, as well as the automations that occur following movement between steps (Multimedia Appendix 1, page 3). For example, when a patient is moved into the “Person Under Investigation” category, a monitoring system is immediately activated, which allows them to report on their symptoms from home using the online patient interface app (Multimedia Appendix 1, page 4). Self-assessments via simple questionnaires allow for repeated and effective monitoring of clinical features.

#### Module 4: Shared, Longitudinal Record

The integrated COVID-19 shared record represents a temporary pandemic longitudinal record. Lack of interoperability between disparate health care systems is an age-old problem. During a time of crisis, we must be less focused on software integrations and more focused on the immediate needs to reduce mortality and morbidity from the terrible onslaught of something like a pandemic. The digital platform provides a lightweight, cloud-based, and secure health record, which serves as the central documentation system for all encounters related to COVID-19.

A new digital workforce can be rapidly onboarded to track patient encounters and supervise patient’s open tasks; a collaborative team can then screen the necessary data to provide care to the patient (Multimedia Appendix 1, page 5). Notably, the entirety of the patient’s medical history and full chart is not integrated into the solution. Given privacy concerns, the emergency record only facilitates the bare minimal data set to provide care in the context of the crisis at hand. Wherever possible, data is collected discretely through the encouragement of patient-generated data using questionnaires and having minimal free-text data entry. The triage and daily monitoring questionnaires only collect data on risk factors for exposure, age, and sex. All data is available to providers at all times and can be accessed on the patient profile. Clinician inputted data can be facilitated through templates with variables, again avoiding free or narrative text to feed immediately accessible data into the integrated analytics engine. As in most patient-provider interactions, the responsibility is on that provider to appropriately triage and follow up with the patient. Currently the platform does not have integration with radiology, but laboratory results are being integrated based on unique patient identifiers by an onboarded digital task force.

#### Module 5: Patient App and Virtual Care

Visits with patients are either going to occur physically or virtually. In the context of the COVID-19 pandemic, it is imperative that, wherever possible, the health care system be able to keep patients at home and away from crowded facilities where their attendance could be responsible for getting infected, spreading infection, or infecting health care workers.

As a component within an integrated system, there is an app available for patients to communicate virtually with clinicians using voice-over Internet Protocol technology, video technology, and live chat (Multimedia Appendix 1, page 6). The readily accessible communication capacity through the app provides an efficient way for health care providers to reach patients on demand. If a symptom tracker is going in a negative direction, a clinician could initiate a virtual visit on demand through this integrated technology.

Patients are triaged according to the programmed branching logic, which is provided by their provincial health authority. In Ontario, for example, patients are triaged into same-day virtual visits with automated daily monitoring or immediate emergency care. This is all done based on patient responses and allows patients to self-isolate or social distance easily. Once a same-day virtual visit is requested, an onboarded provider is notified and takes over the patient’s management.

#### Module 6: Integrated Analytics

All data that is collected through the other modules are fed into an analytics engine, which allows for the creation of ad-hoc data visualizations and exportable reports that provide real time data (Multimedia Appendix 1, page 7). Data can be presented in a multitude of fashions such as on aggregate population levels or separated into cohorts. By integrating analytics in this fashion, a tremendous amount of time is saved from the typical data processing that health care systems are used to. Data is not required to be exported out of the platform for analysis. The data pipeline provides the “air traffic control” style views that administrators need to make better decisions, identifying system delays and assessing outcomes.

### How This Crisis Management Platform Addresses Our Current System Delivery Flaws

A number of problems identified in current health care and crises management systems are addressed using the proposed system, as summarized in the following bullet points:

- Triage and surveillance happen automatically, “en masse,” without absorbing resources and in a scalable and standardized manner.
- Care delivered on a virtual platform is more efficient for patient and provider. Physicians will, therefore, have more time to apply thought to the specific nuances of the problem at hand.
- With a virtual model of screening and daily monitoring via questionnaire with automatic alerts, it is possible to manage the majority of patients in their own homes, addressing patient health as well as source control. The risk to health care workers will be less too.
- Standardization is addressed with questionnaires that are continuously reviewed and adapted if need be to help compare findings and improve the quality of surveillance.
- Patient engagement is inherently improved, as they have a critical role and can become an active part of the solution.
- Built-in analysis is automated, and audit processes will become part of care as usual. An important aspect is patient reported outcomes that will also become routinely generated and tracked longitudinally, identifying continuous opportunities for quality improvement.
- It can provide referral for health concerns related to COVID-19.

### Privacy and Security

As with any digital health system involving the capture of large amounts of personal health information, privacy concerns must be considered. This is particularly true in this effort, where a large number of clinical users will be accessing a shared system. Privacy concerns will be dealt by:

- Technical considerations such as per user-level access restrictions and control, ability to generate audit logs, and ability to track unusual access patterns
- Regulatory considerations such as having completed privacy impact assessments on the platform
- Process considerations such as centralized control over the ability to add new users and control permissions, as well as privacy training
- Advisory considerations, including engagement with privacy expertise both on the technology and regional front

Security is paramount when discussing scalable systems in health care. The Google Cloud Platform has been employed to provide several layers of encryption to protect customer data at rest. Multiple third-party security assessments have been completed, and the development team has more than 10 years of experience deploying enterprise projects. In addition, a dedicated team works to ensure security processes are tested and remain updated.

## Discussion

### Principal Findings

Public health crises are the moments of truth on a systems level. They disclose problems but also provide learning and opportunities for innovation and necessary disruptive changes. Mistakes happen and action driven through anxiety will also occur. Although some flexibility is important for the collaborative efforts of self-organizing and cross-functional teams, rapid and flexible responses to problems brought on by a lack of governance can make the difference [17].

Web-based communication and online resources can be disruptive because they have the potential of changing the whole process of care delivery, from facilitating access (much easier in remote locations) and engaging with patients in an ongoing way (asynchronous communication, motivational enhancement, gamification) to offer quality care with no direct professional involvement (online health promotion, psychoeducation, psychotherapy) [12,13]. These changes build the capacity to increase coverage and improve quality [12,13].

It is clear that telehealth has contributed positively to previous crises; mobile app tracking during the Ebola crisis and video conferencing during the severe acute respiratory syndrome outbreak are examples of this [2]. Similarly, after the Haiti earthquake, a mobile health information technology (IT) platform with over 600 patient entries enabled adequate triaging and improved continuity of care and provider hand offs [18]. For COVID-19, virtual heath care companies across the world have enabled secure communication between providers and patients [2]. In China, the Emergency Telemedicine Consultation System enabled remote monitoring of 63 severe cases and 591 patients with mild cases of respiratory infections, of which 420 cases were cured and discharged [19]. This tool improved outcomes by effectively collecting and evaluating patient health data and efficiently bringing together specialists from different clinical disciplines, thereby avoiding shortages of resources and allowing for comprehensive assessment and treatment [19,20]. The CMP, like many other tools, supports and enables communication and coordination between health care’s different disciplines.

Implementation studies are required to confirm whether the CMP can improve outcomes and help deal with limited resources. However, the concept offers some realistic expectations. The online triage, which includes a self-assessment, is serving a big group of concerned citizens in the lineup for testing—over 13,479 patients had been triaged as of March 23, 2020. This is also keeping them away from centralized services that offer medical advice, such as phone lines, which have been notorious for long wait times [21]. Additionally, the self-tracking of symptoms supports health professionals later, as it documents the clinical trajectory of the patient. Finally, the first implementation responses from physicians have been positive, with over 350 medical doctors onboarded in the several weeks it has been active.

The system’s use is only expected to increase [22]. Health systems have seen their share of virtual visits grow from less than 1% of all visits (in-person and virtual) to 70% within a 4-week period [22]. This is understandable, given the fact that virtual care facilitates the avoidance of physical contact and prevents the potential transmission of infection [19]. Among health professionals, this is especially critical: almost 10% of all infected cases in Italy have been health care workers [23]. Additionally, physicians are embracing virtual care, as it allows them to monitor and treat patients at a much larger scale and with little risk of infection [20]. On the patient side, acceptance of online treatment has been shown to be increasing, especially if the process is transparent and directly engages them in the management of their care [24,25]. This solution gives access control to the patients, who may use this information while still allowing the most responsible physician to have oversight and coordinate the process.

The rapid deployment of the solution does not address its integration into existing electronic medical record (EMR) systems (whether hospital- or clinic-based). In fact, the digital platform, which has pre-existing Health Level 7– and Fast Healthcare Interoperability Resources–based architecture, is not able to easily integrate with the existing systems at the velocity that would be required for deployment in the context of a crisis [26,27]. The restrictions are not based on developer or technology constraints, but rather the lack of any expedited process such as privacy impact assessments for third-party integrations into central or core health information systems. Despite billions and billions of dollars of investment in health IT infrastructure, the existing heath information systems are rigid, disparate, and fragmented, an even more intolerable state of affairs in a time of crisis [21,28]. In effect, the development of a separate independent solution for the epidemic, despite the fact that there are already established EMR systems, is due to the inability of the current solution to address the described problems with triaging, tracking, and disease management due to COVID-19 [29]. This is an issue of critical importance given the fact that the Canadian government provided IBM, the multinational technology company, with a multimillion-dollar budget to develop a tracking system as part of epidemic preparedness, which turned out not to be functional [28]. Finally, all other solutions interact with health care professionals without providing users with direct access to their own data. However, patient portals tethered to EMR systems improve patient engagement and health outcomes [8]. Therefore, developing a separate standalone platform was a more feasible approach to integrating an existing system, given the time pressure and pre-existing structural and regulatory problems on a system level.

It is apparent that the deployment of new digital systems may fragment and isolate care pathways, even further than they already are [7,19]. One way the CMP addresses this is by identifying patients that are attached versus unattached to primary care during their registration. Attached patients are put into a pathway that makes it easy for their own primary care provider to access the system and manage their care, where available. Unattached patients are seen by a virtual physician workforce that is acting more in a walk-in style model during this time of crisis.

### Limitations

There are limitations to this approach. First, the platform was purposefully deployed in a standalone fashion, not integrated through any meaningful application program interface (API) into existing infrastructure. This was done to avoid delays related to developing said APIs and other issues of compatibility but could be reversed if and when that becomes desirable. Due to the lack of such integrations, certain actions like hospitalization of the patient require manual data reconciliation as opposed to the ideal automated state triggered through software. Second, although online technology is quite ubiquitous in most societies, those that are most vulnerable and marginalized in society are often the ones who have no access. Although proxy people can facilitate technology on their behalf, it is important to not isolate them or worsen their outcomes through further neglect. Voice-based systems (notably scaled down) need to be maintained to serve these patients. Third, rapid privacy impact assessments are needed in a time of crisis. There is no defined methodology, and a rapid rollout of technology could compromise patient privacy if not governed in an intelligent and streamlined fashion.

### Assets of the Crisis Management Platform

Based on correspondences with the implementation team including providers, decision makers, researchers, and health care workers, several specific theoretical advantages can be conceptualized.

The assets from a health system’s perspective are:

- Integrated population health tool that can efficiently track patients and identify what's going on, where it is happening, and how to reduce the spread in real time
- Patients can enter data points themselves, and doctors have the opportunity to confirm the data inputted and can then apply it to the patient’s profile. This will attempt to increase the efficiency of our health care system in the context of a surge in volume and allows patients to become a part of the solution.
- The system can send mass questionnaires to the whole patient load, with the ability to modify different filters to target specific patient demographics. When those questionnaires come back answered, we can also screen for specific things and send specific individuals different questionnaires depending on different risks, which points to efficiency and scalability.
- Positive test tracking: data inputted from any active lab results in the system or patient data points flagged in a chart as being positive goes into an analytic visualization that allows users to see how tests change overtime and can use heatmaps and report by age, gender, sex, etc, providing automatic analysis.

The assets from a health care worker’s perspective are:

- The questionnaire builder can produce any type of branching logic-based questionnaire with a way to distribute these questionnaires directly to patients using smartphones through text messaging and email. As this screening and surveillance program grows, we would build a few different collections of these screening tools, which would be available and distributable. Crises require logical thought and flow. We need a thought-out structure to work efficiently, and questionnaires provide this.
- The custom data system can produce a data dictionary from answers, organically building a registry of patients with data coming directly from the patients themselves. Mandatory fields ensure capturing of the most important data points and limit missing data.
- Full workflow management system: the dashboard allows providers to create pathways for patients, moving them across the different phases, which allows them to actually track the activity live. This system also tracks all interactions so that problems in the supply chain can be identified. It also tracks the amount of time people spend on these individual stages, so from a population and community perspective, this allows for quality assurance or allows us to understand the metrics around the different stages of care.

The assets from a patient’s perspective are:

- Booking and registration: patients can self-register into shared EMRs and self-book appointments with available physicians. Prescreening questionnaires can classify their risk based on their answers and prioritize them in the booking queue. An alert system for the health care professionals becomes activated if the patient is at a very high risk and initiates immediate action.
- Push notifications for confirmation and reminders make the communication with providers convenient and easy. Automatically queued actions and questionnaires are based on transitions and assigned tasks. Self-management strategies such as references and tools can be automatically queued (eg, being transitioned to quarantine could automatically queue videos for hand-washing and daily questionnaires for symptoms). Patients can fill in an active daily monitoring questionnaire, which updates us on symptom progression, tracks whether the patient is getting worse, or whether they need to get to a hospital, etc. Compliance to these tasks are tracked to engage with patients and support them with appropriate measures.
- The system supports all ways of communication (chat, video, secure message) with physicians, and health institutions try to make access as easy as possible.

Web-based solutions can be a significant asset in the crisis response to a public health threat [30]. Best case would be to have an appropriate system in place with the necessary functionalities. The slowest part is the decision making; after that, solutions can be developed and built up to work quickly. The current solution is an example, which needs further research. It provides important learnings about process and outcomes for the future of crisis management. The next steps include evaluating its implementation within the community and adapting the system in response to the needs of the health care system and to the evolving crisis.

### Conclusion

A paradigm shift toward eHealth requires a change in mindset, but many systemic health problems can be better managed in this manner—COVID-19 just happens to be a perfect example of such a change. The pandemic has generated a sense of urgency that will allow the adoption of innovation without the logistical barriers and path dependencies that we have become accustomed to.

Web-based solutions like the CMP can fill a critical gap for health care resource allocation. It demonstrates the potential effectiveness of patient-centered, web-based solutions built on outcome-driven architecture, which needs to be proven by further evaluation and research. Data- and web-based approaches are key to evidence-driven oversight and management of health care systems during public health crises.

## Appendix

Multimedia Appendix 1 Crisis Management Platform functionalities.

## Abbreviations

API: application program interface
CMP: Crisis Management Platform
COVID-19: coronavirus disease
EMR: electronic medical record
IT: information technology
