# Supplementary material for: Emergency Response to COVID-19 in Canada: Platform Development and Implementation for eHealth in Crisis Management
Source: JMIR Public Health Surveill. 2020 May 15;6(2):e18995. doi: 10.2196/18995 (PMC7236607; doi:10.2196/18995)
Supplement: Multimedia Appendix 1 [file publichealth_v6i2e18995_app1.pdf]

CENTRAL INTAKE + TRIAGE

Single Access Point for Patients, Caregivers, LTC, GP's, and 811 Nurses

BRITISH COLUMBIA

Welcome to the COVID-19 Screener.

This tool is your central access point to access provincial services focused on conquering the COVID-19 pandemic. Please answer to to the best of your ability for you, or the dependent individual you are completing the tool for.

**** SERVICE IS ONLY AVAILABLE TO THE CITIZENS OF BC ****

Start press **Enter** ↵

3 → Check all of the following symptoms you may have:

Choose as many as you like

- A Fever* ✓
- B Cough* ✓
- C Shortness of breath at rest*
- D Chest pain
- E Muscle ache / fatigue* ✓
- F Headache
- G Runny nose
- H Sore throat
- I Diarrhea
- J Nausea and vomiting
- K None of the above

OK ✓ press **Enter** ↵

Answers are scored to assign patient into high, med, low risk category for resource allocation.

deflect inappropriate referrals

You don't need to be tested for COVID-19. Due to limited supply of tests, we are only testing individuals who are actively symptomatic with cold.

again press **Enter** ↵

Assure that high risk persons are dealt with first!

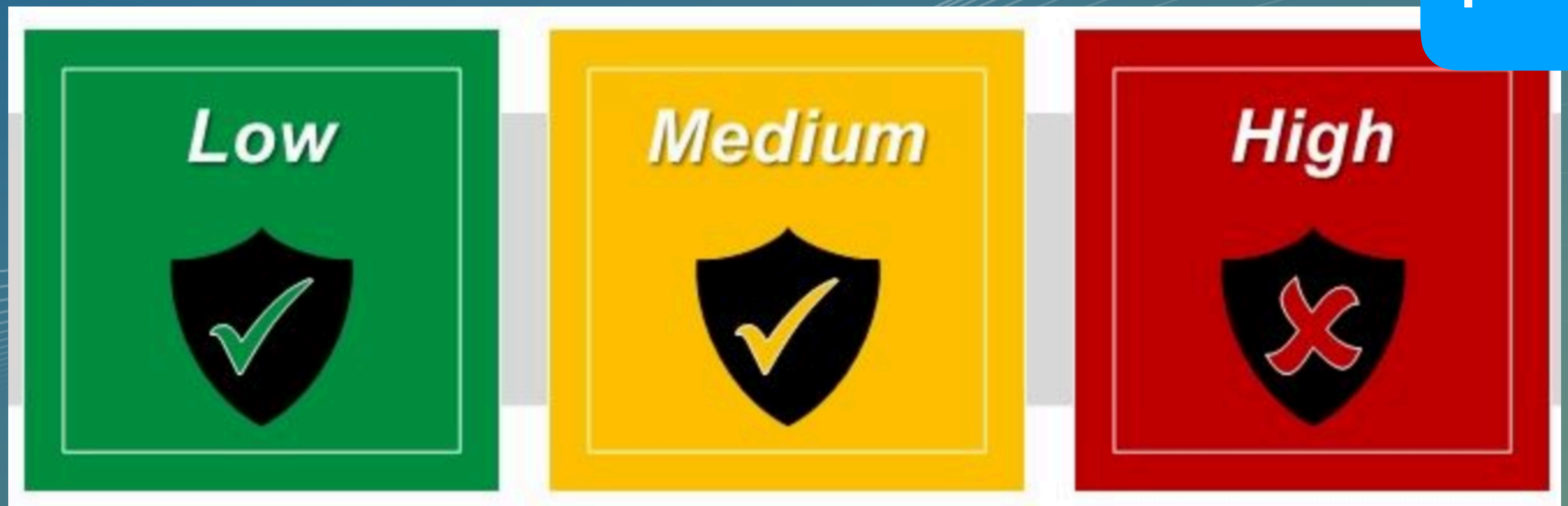

BOOKING + REGISTRATION

Patients (or proxy) can register + self-book.
Based on central intake, they are already risk classified and high risk gets first priority on booking queue.

Supports SMS and/or e-mail notifications for confirmation + reminders.

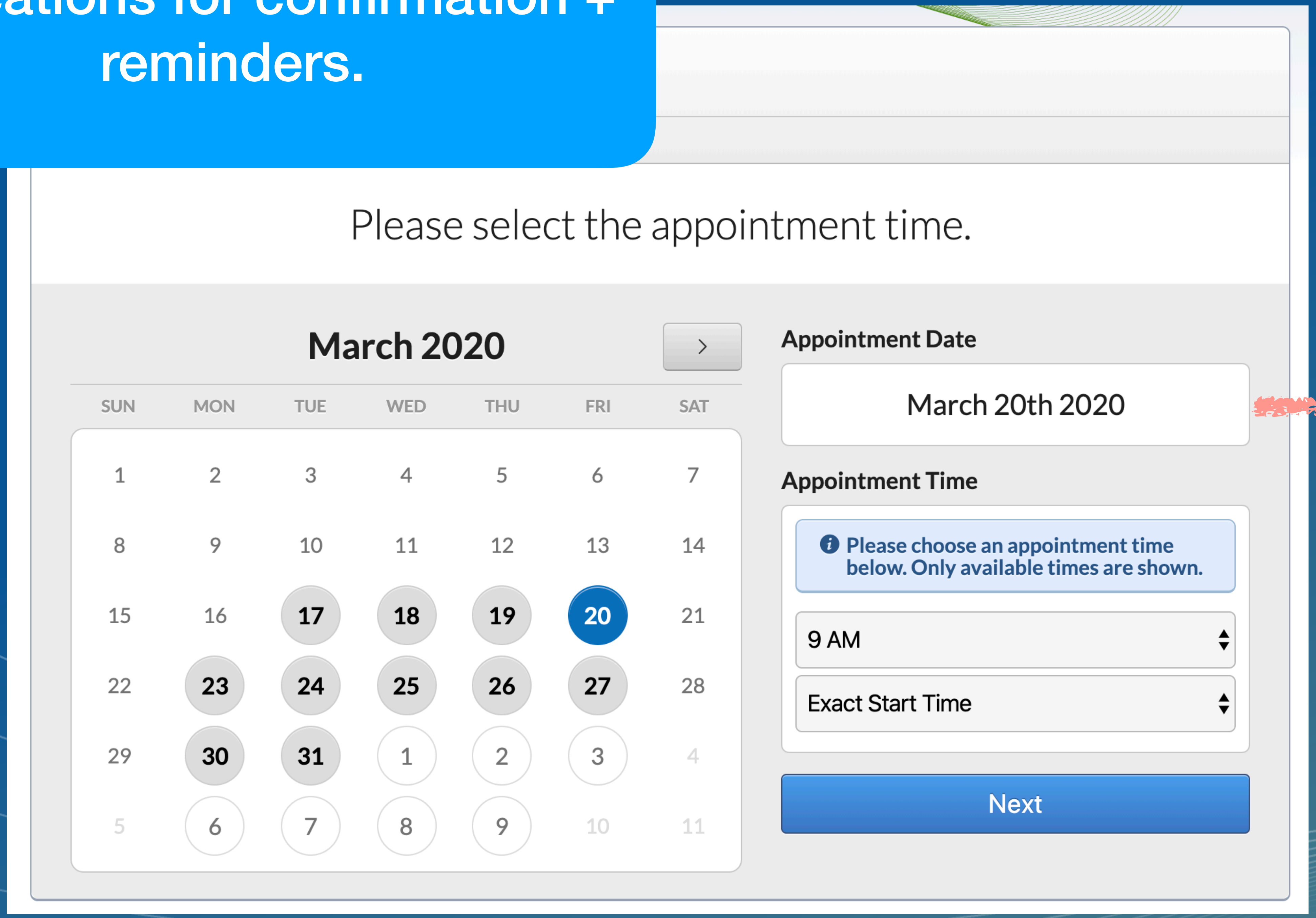

Self register into shared EHR.

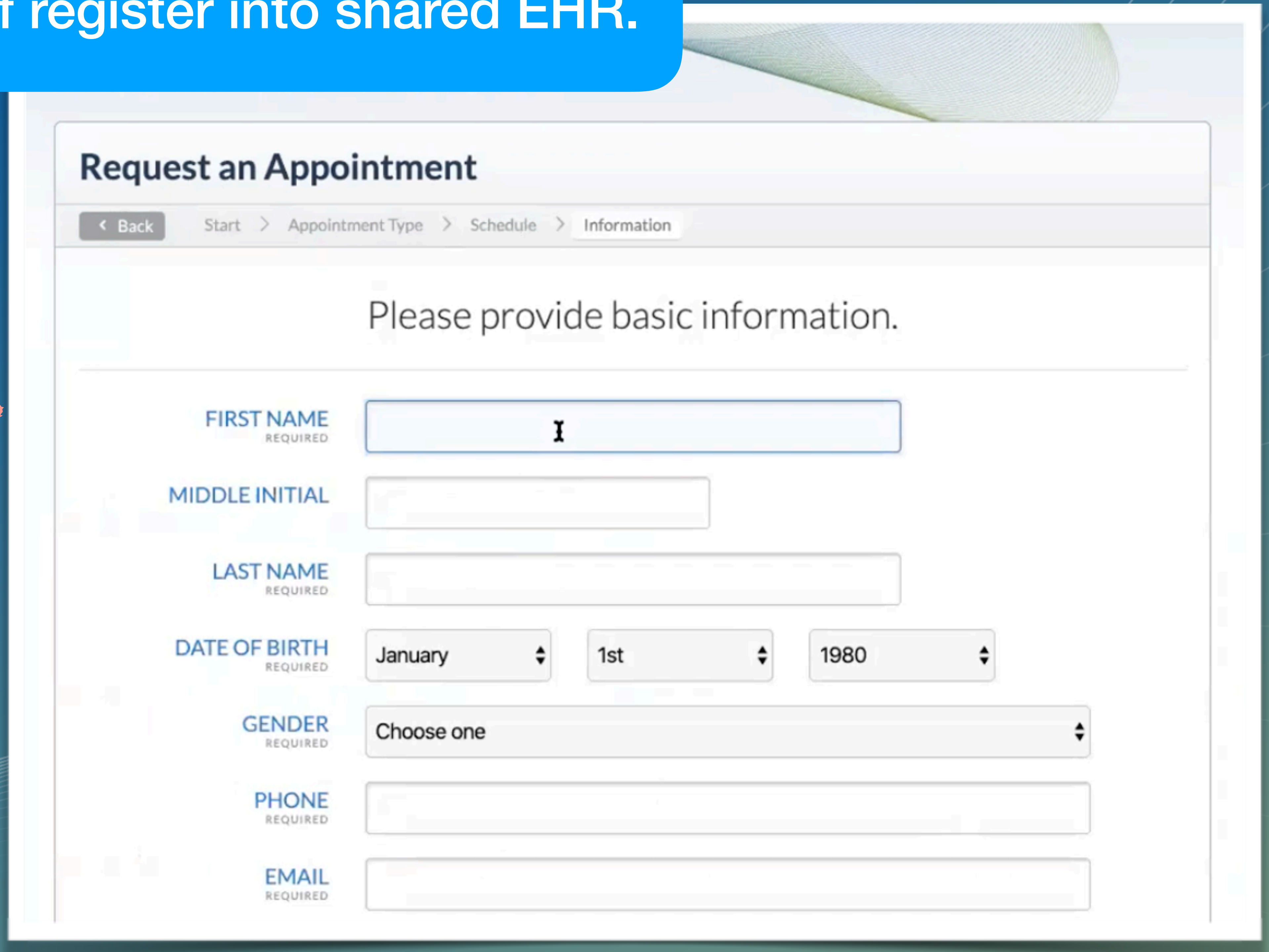

FLOW TRACKER

After registration - patient is immediately tracked through a workflow board with built-in rules logic.

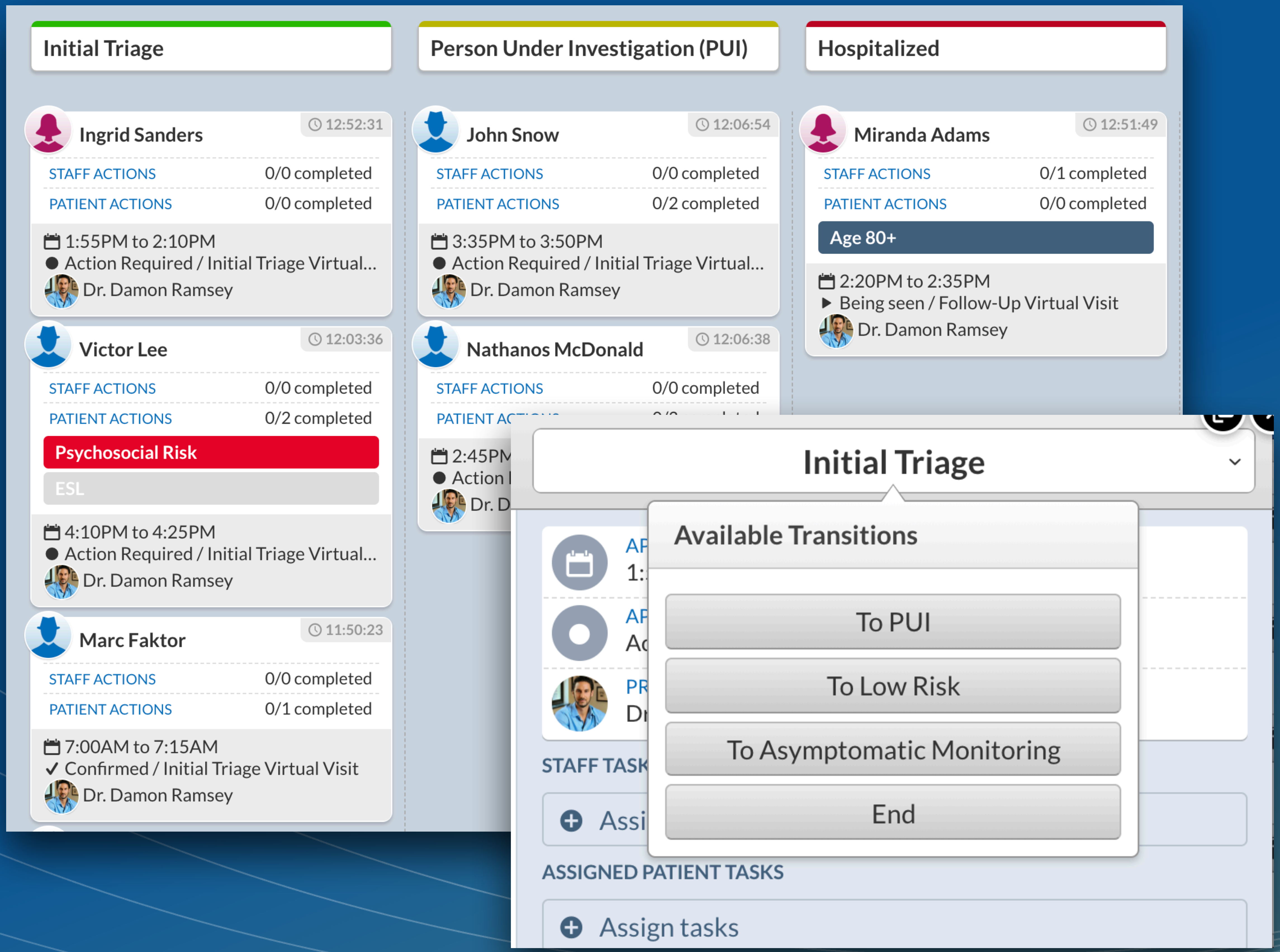

- **Keep patients organized in a central, filterable source**
- **Transition patients through pathways based on pre-defined logic**
- **Automatically queue actions based on transitions to support busy staff**
- **With coupled mobile app, can communicate (chat, video, secure message) from single spot**

Patients can be assigned tasks Compliance is tracked

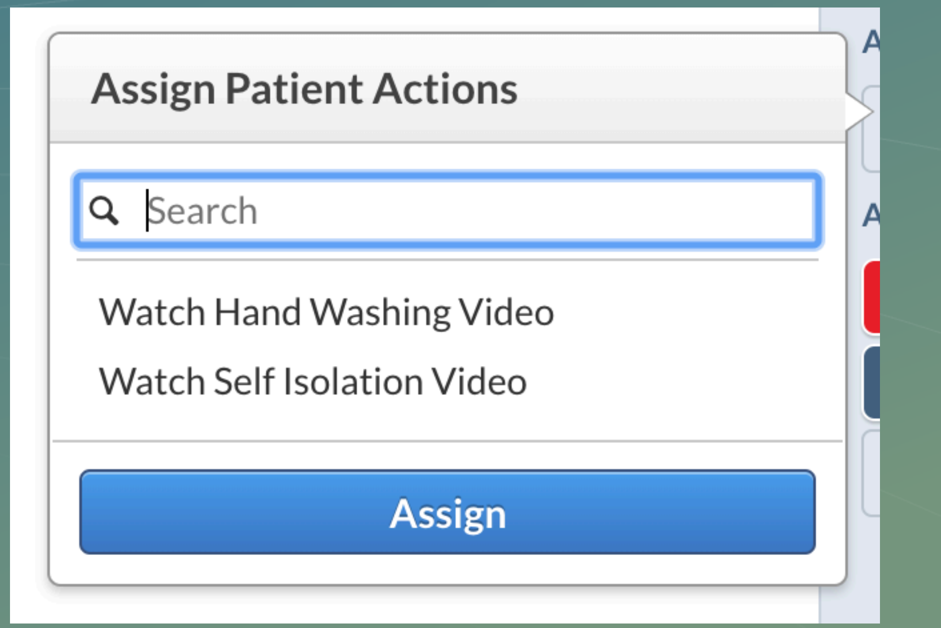

SYMPTOM MONITORING + ALERTS

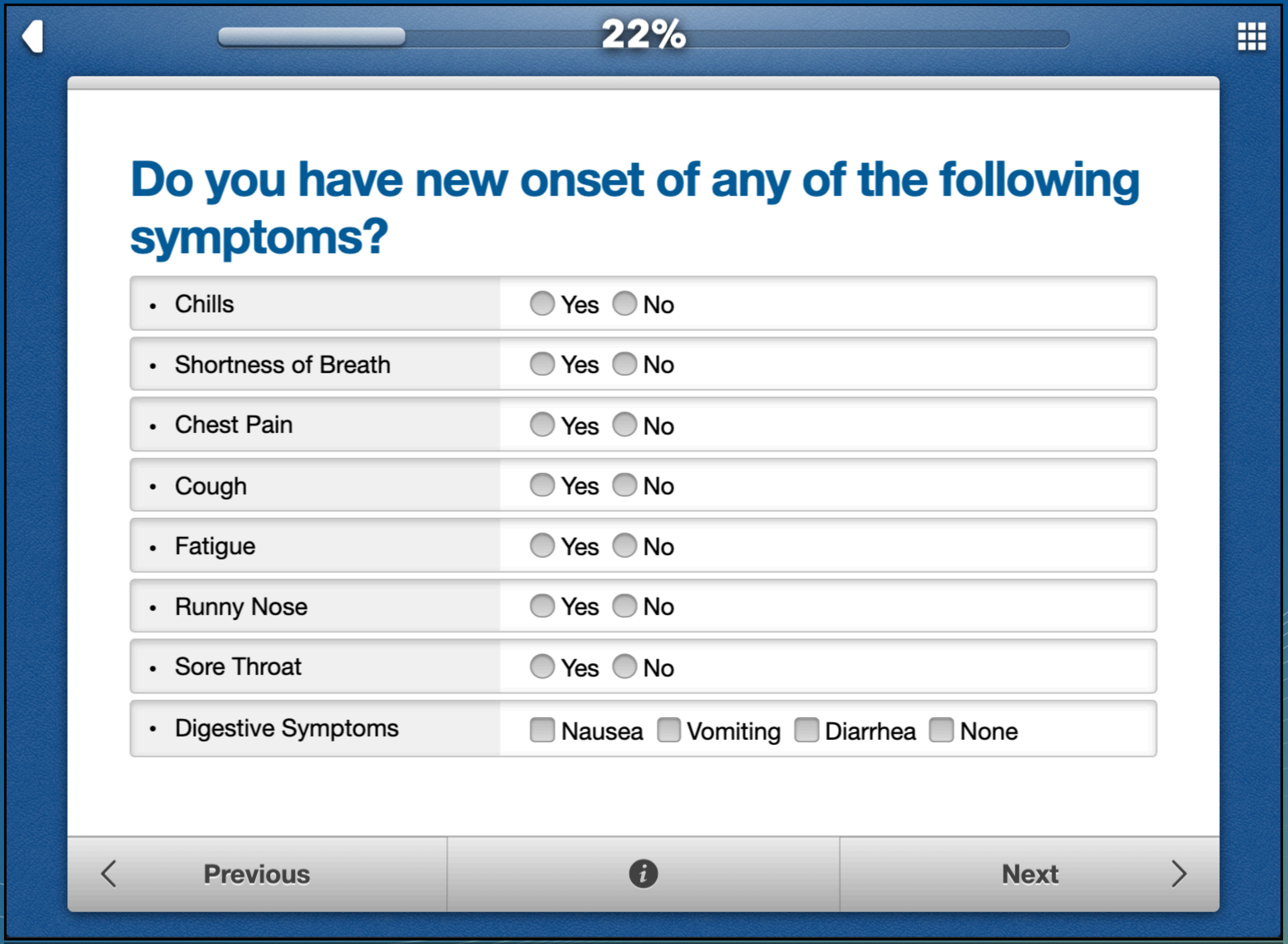

ALL ENCOUNTERS TRACKED

PATIENTS MONITORING THROUGH AUTOMATED SCHEDULED QUESTIONNAIRES

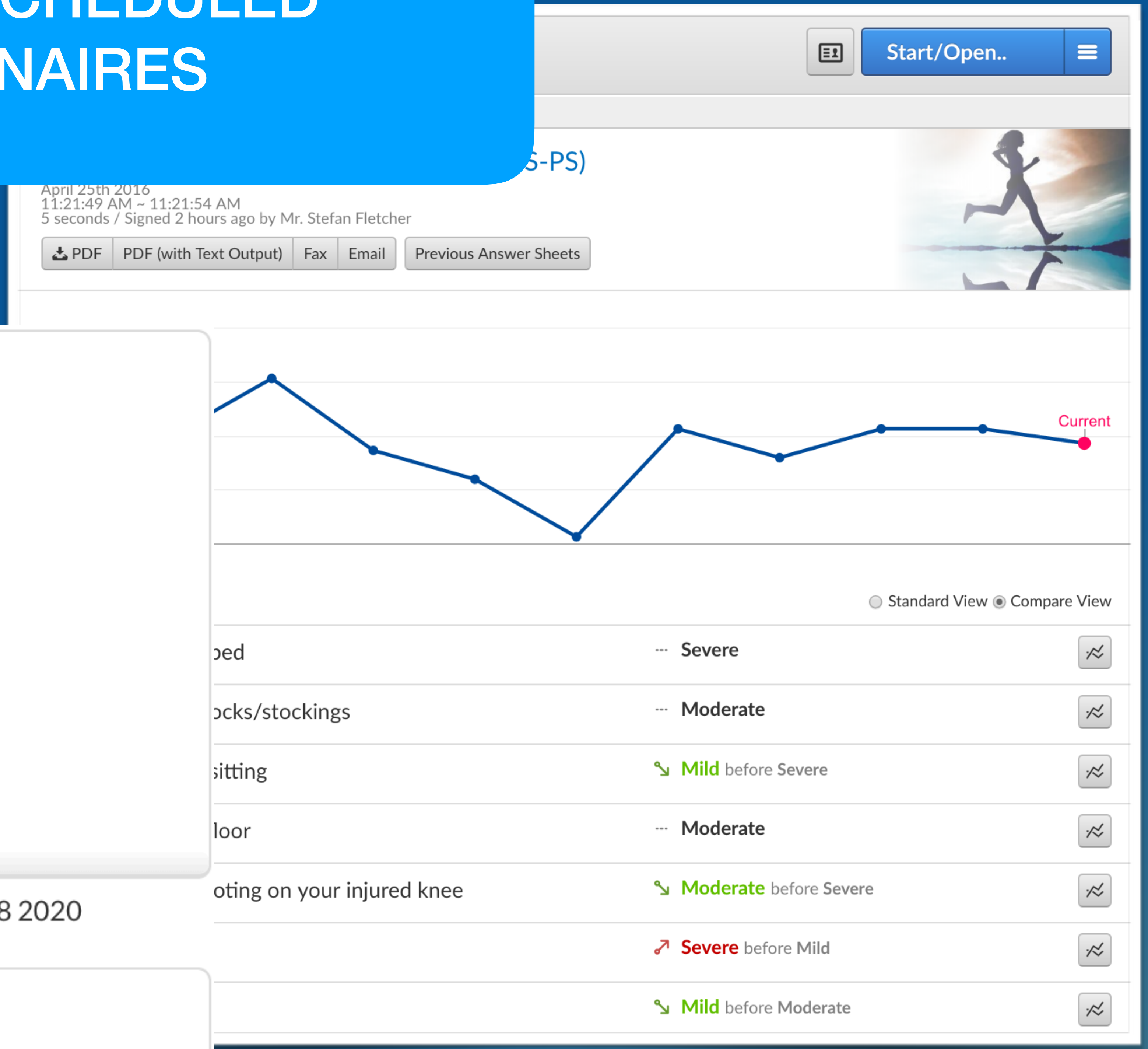

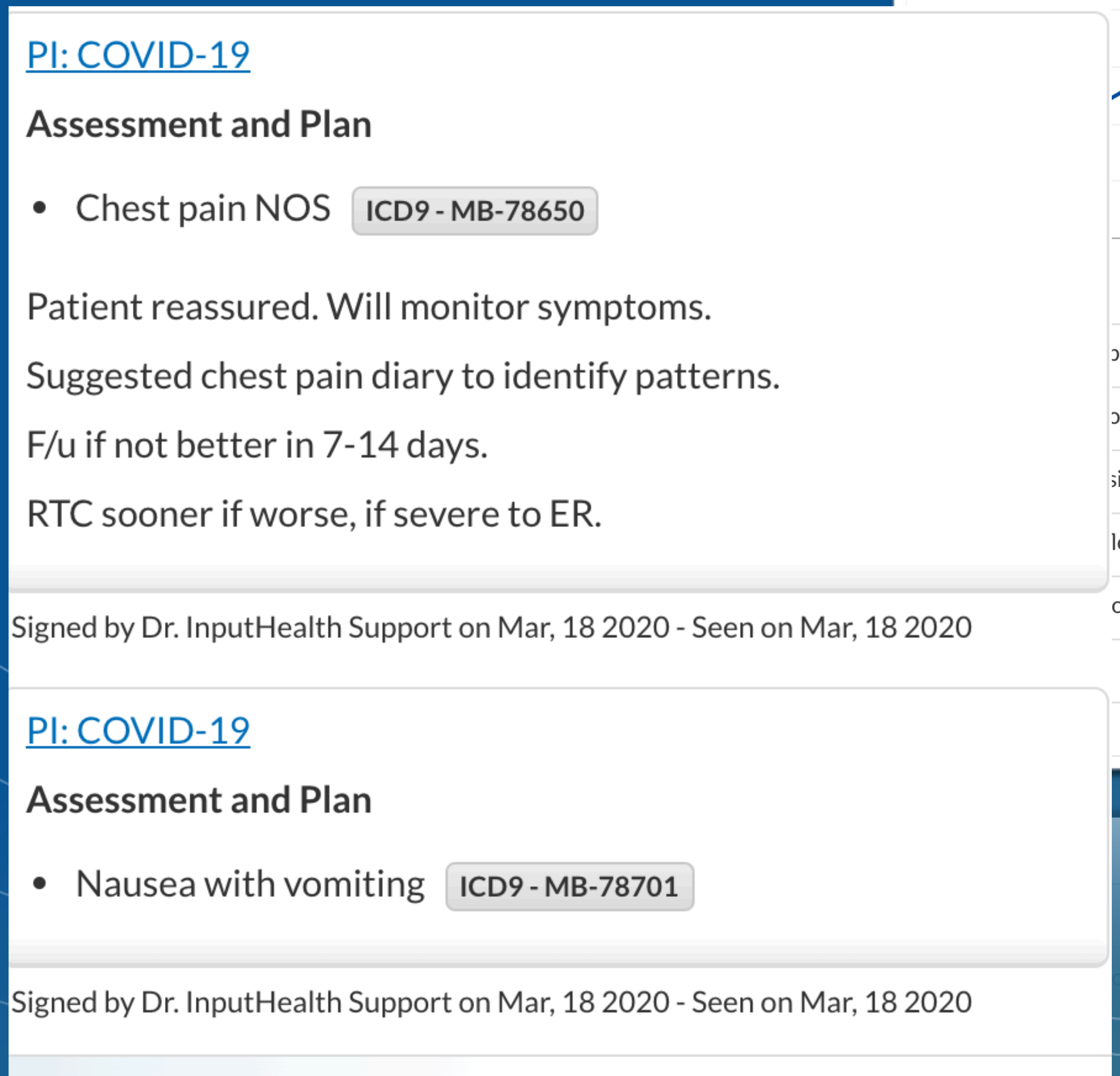

PI: COVID-19

Assessment and Plan

- Chest pain NOS ICD9 - MB-78650

Patient reassured. Will monitor symptoms.

Suggested chest pain diary to identify patterns.

F/u if not better in 7-14 days.

RTC sooner if worse, if severe to ER.

Signed by Dr. InputHealth Support on Mar, 18 2020 - Seen on Mar, 18 2020

PI: COVID-19

Assessment and Plan

- Nausea with vomiting ICD9 - MB-78701

Signed by Dr. InputHealth Support on Mar, 18 2020 - Seen on Mar, 18 2020

- **Phone, video, in-person all in one central source**
- **Templates for data standardization**
- **Shareable easily**
- **Discrete data is captured for reporting purposes**
- **Connected directly with MSP TELEPLAN FOR BILLING**

VIRTUAL CLINIC + PATIENT APP

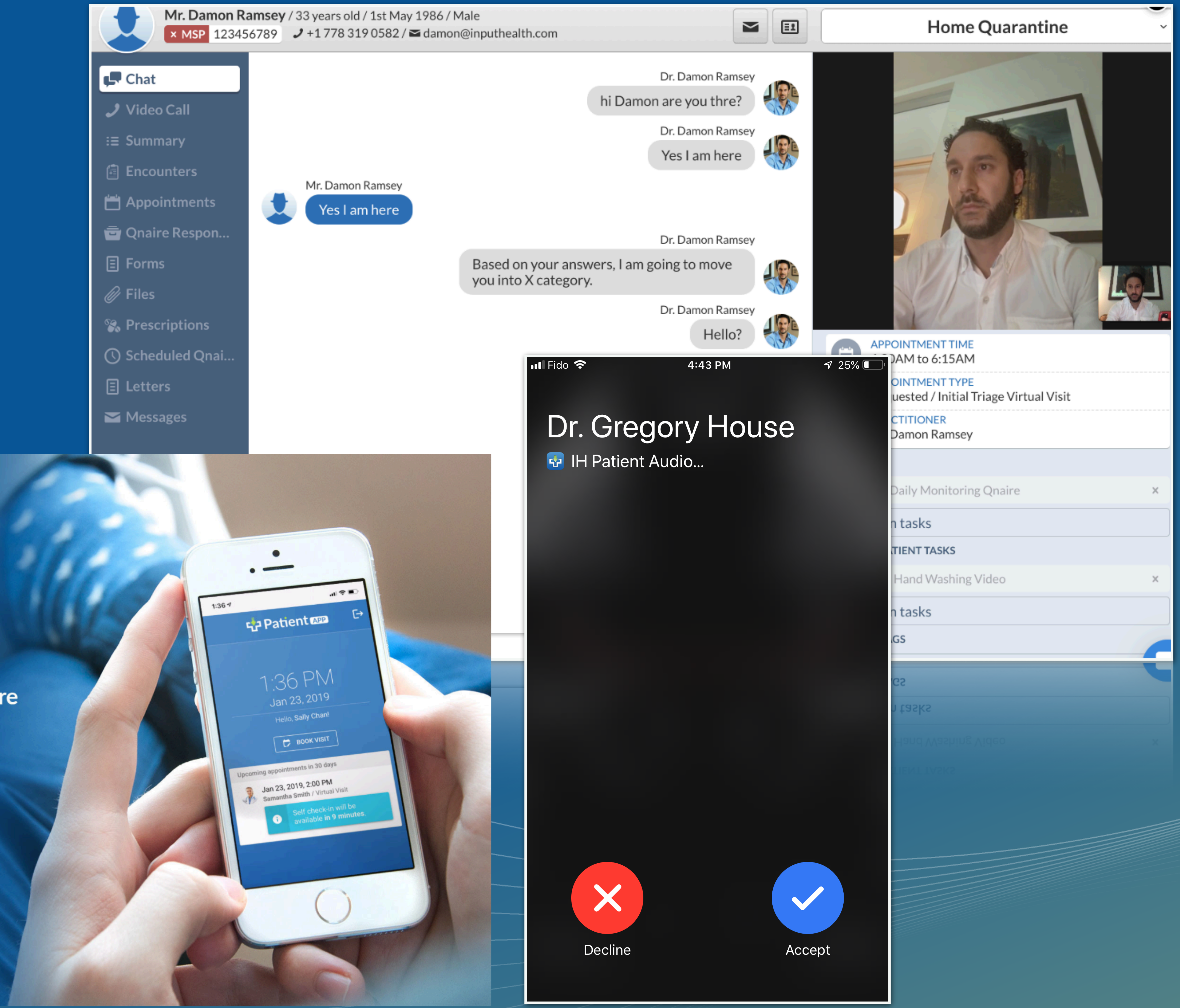

- Video/audio call directly from the platform - Zoom integration TBD
- Live chat with patients on-demand as well
- Works through a mobile app on the patient side **(can be built + branded for BC and submitted to Apple + Android app stores this week)**
- **COVID-19?**

“There’s an app for that.”

INTEGRATED ANALYTICS ENGINE

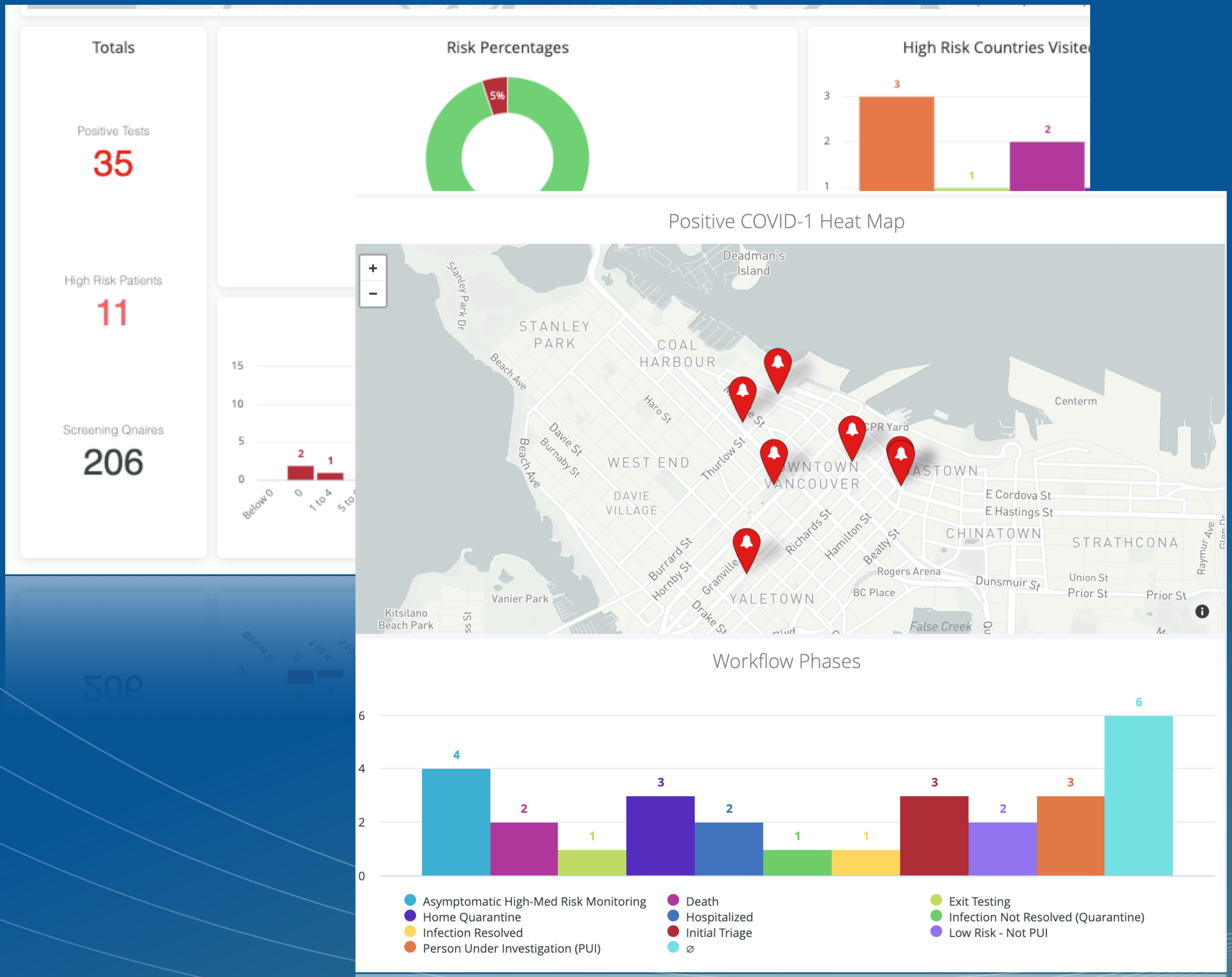

- **LIVE, REAL-TIME data visualization**
- **Exportable data on demand**
- **Can create custom reporting on-demand with automatic alerts and distribution**
